# Service Integration Across Sectors in Europe: Literature and Practice

**DOI:** 10.5334/ijic.3107

**Published:** 2018-04-19

**Authors:** Sarah van Duijn, Nick Zonneveld, Alfonso Lara Montero, Mirella Minkman, Henk Nies

**Affiliations:** 1 Departement of Organization Sciences, Vrije Universiteit Amsterdam, De Boelelaan 1105, 1081HV, Amsterdam, NL; 2 Vilans, Centre of Expertise for Long-term Care, Catharijnesingel 47, 3511GC, Utrecht, NL; 3 University of Tilburg/TIAS, Tilburg, NL; 4 European Social Network, 125 Queens Road, Brighton BN1 3WB, UK; 5 Vilans and Department of Organization Sciences, Centre of Expertise for Long-term Care, NL

**Keywords:** Service integration, collaboration, integrated care, social services, cross-sector service delivery

## Abstract

**Introduction:**

To meet the needs of vulnerable people, the integration of services across different sectors is important. This paper presents a preliminary review of service integration across sectors in Europe. Examples of service integration between social services, health, employment and/or education were studied. A further aim of the study was to improve conceptual clarity regarding service integration across sectors, using Minkman’s Developmental Model for Integrated Care (DMIC) as an analytical framework.

**Methods:**

The study methods comprised a literature review (34 articles) and a survey of practice examples across Europe (44 practices). This paper is based on a more comprehensive study published in 2016.

**Results:**

The study demonstrates that although the focus of integration across sectors is often on social services and health care, other arrangements are also frequently in place. The review shows that integration may be either tailored to a particular target group or designed for communities in general. Although systems to monitor and evaluate social service integration are often present, they are not yet fully developed. The study also highlights the importance of good leadership and organizational support in integrated service delivery.

**Discussion:**

The study shows that the DMIC can work as a conceptual framework for the analysis of service integration across sectors. However, as this is an exploratory study, further in-depth case studies are required to deepen our understanding of the processes involved in service integration across sectors.

## Introduction

Although integration across sectors is taking place throughout Europe, there is still limited understanding of the working practices across these sectoral boundaries [1]. In this review, integration is interpreted broadly and is defined as:

“independent, yet interconnected sectors working together to better meet the needs of consumers and to improve the quality and effectiveness of service provision” [1, p. 17].

There are different forms of integration, ranging along a continuum from linkage to full integration, which aim to achieve coherence and synergy between organizations and professionals as well as enhanced quality and efficiency of delivery [1,2,3]. This review takes social services as a starting point and focuses on cases designed to foster the well-being of vulnerable people with a wide range of problems affecting various domains in their lives. The social domain is considered to have a central role in supporting people to manage their lives and deal with ‘opportunities and limitations’ [4,5]. The need for robust social services is also increasing because of current policies aimed at keeping vulnerable people longer in the community instead of in institutional care [6]. Cross-sector service integration, however, is not easy to accomplish, given the complexity of collaboration across different organizations and sectors [7,8].

This paper describes the current state of service integration across sectors in the provision of social services in Europe. It explores what happens at ‘the boundaries’ between sectors [1], for instance, the communication, coordination, and collaboration that takes place across different organizations as well as across sectors. The lack of conceptual clarity regarding integration across sectors may inhibit scientific understanding needed to underpin policy decisions and conversations among researchers, policy makers, and practitioners [1]. The use of a conceptual model can help to clarify the process and structure of service integration across sectors. This paper therefore also explores whether and how integration can be understood across sectors by applying similar concepts, processes and mechanisms used within the framework of integration in health care. The aim of this paper is to explore whether practices of integration across sectors – in this case social services, health, employment and education – can be analysed according to the key elements of the Development Model for Integrated Care (DMIC) [9]. This model has been developed and validated in several fields within the health sector. It has the advantage of being generic, i.e. it does not focus on a specific target group or type of support or care, but also takes inter-organizational and inter-professional processes into consideration. Also, the DMIC has been used in several international projects which have shown it to be applicable in different contexts.

## Theory and methods

Research was carried out to identify current developments in the delivery of integrated social services across Europe. The integration between social services and one or more of the sectors: health care, employment or education was investigated. The study ‘Integrated social services in Europe’ consisted of an overview of recent developments in social welfare, a summary of legislation and policy frameworks, a literature review, and an assessment of practices in various European countries [10]. This paper presents the results of the literature and practice reviews.

The DMIC was used as the initial analytical framework to help identify seven questions that formed the basis of the literature and practice reviews. This validated model was selected because of its generic character and because it permits both a conceptual approach and a practical description of activities that are relevant for integrating services. Also, its current use in a number of international projects has shown it to be applicable in different contexts. Other models, such as the Rainbow Model may also be of value because of their focus on integration, however they do not offer well-defined activities in addition to a conceptual framework [11]. The (Expanded) Chronic Care model has been adopted widely on an international scale, and although it is a conceptual model with relatively well-defined activities, the focus is on people with chronic conditions and population health [12,13].

**Figure 1:**
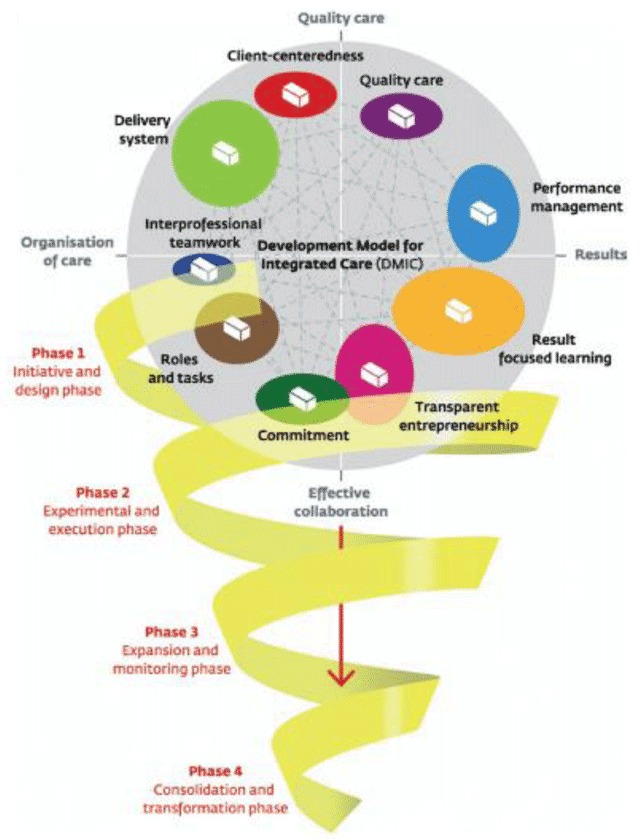
Development Model of Integrated Care (DMIC) [9].

The DMIC model (Figure 1) describes the most relevant activities for service integration [9]. This model comprises 89 elements, which are described as activities relevant to the development and implementation of integrated care (e.g. the provision of understandable and person-centred information). According to the DMIC, the extent of practice integration correlates with the number of elements of integrated services identified The activities are grouped into nine clusters as follows:

1. Person-centeredness (originally client-centeredness): the development of integrated services and information flow which is tailored to a particular individual or target group.
2. Delivery system: the chain of logistics pertaining to the practice. This cluster focuses on the mechanisms and processes that are in place to streamline the provision of the service.
3. Performance management: the measurement and analysis of the outcomes of the service(s) delivered and subsequent feedback to manage and improve performance of delivery.
4. Quality care: the quality of design of the practice including elements such as adherence to evidence-based guidelines standards, and the needs and preferences of service users. In this study, the clusters for performance management and quality care have been combined since they both address measurement, standardization and evidence.
5. Result-focused learning: the presence of a learning climate in the practice which encourages continuous improvement.
6. Inter-professional teamwork: inter-disciplinary teamwork for a defined target group achieved through the collaboration of professionals working across the delivery chain of the service.
7. Roles and tasks: effective collaboration at all levels based on clear definitions of individual expertise, roles, and tasks. Given the close relationship between inter-professional teamwork and roles and tasks, the findings from both clusters have been combined.
8. Commitment: collaborative working practices based on clear goals and awareness of inter-dependencies and domains.
9. Transparent entrepreneurship: capacity for innovation, leadership responsibilities, and financial agreements regarding integrated care.

**Figure 2:**
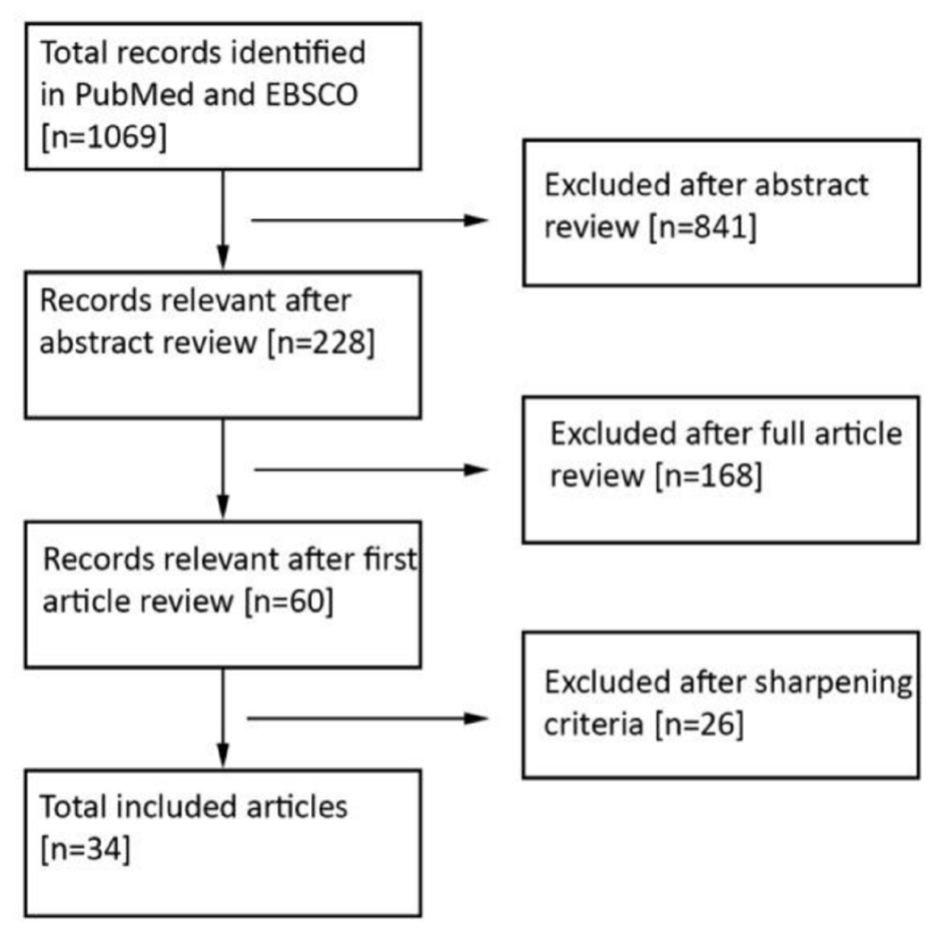
Flowchart literature review.

Early in 2015, the databases ‘PubMed’ and ‘EBSCO’ were searched for European studies on social service integration using combinations of search terms such as ‘integration,’ ‘integrated care,’ and ‘shared social services’ (see Table 1). The initial search was based on review studies and evaluations from examples of integration in Europe. The aim was not to carry out an exhaustive review, but to identify literature in which the processes and mechanisms behind service integration, and social services in particular, were addressed. As the flowchart indicates (see Figure 2), the searches identified 60 publications which presented (review) studies or narratives on social service integration and these were included in the analysis for study ‘Integrated social services in Europe.’ Since the focus of the present paper is the analysis of empirical studies of service integration, the inclusion criteria were narrowed to exclude non-peer reviewed and review studies from the initial search results. The studies included in this paper therefore met the following criteria: (1) European examples, (2) cross-sectoral integration that includes the social sector, (3) studies published in the period 2010–2015, (4) studies presenting empirical evidence based on actual cases, (5) peer-reviewed publications in English. The 34 articles included in this review were coded in MaxQDA and analysed according to the clusters of the DMIC.

**Table 1:** Search term combinations.

Database	Search term

Pubmed	Shared Social Services & Health & Europe
EBSCO	Social services & joint working
EBSCO	Social services & education & partnerships
EBSCO	Social services & employment & whole system
EBSCO	Social services & holistic
Pubmed	Shared Social Services & Education & Europe
EBSCO	Public services & integration
EBSCO	Social services & employment & intersectoral
Pubmed	Integrated services & Social Sector & Europe
EBSCO	Social services & education & whole system
Pubmed	Social services & integrated working & public authorities
Pubmed	Social services & integrated services & Europe
EBSCO	Social services & education & integrated working
EBSCO	Social services & education & intersectoral
EBSCO	Social services & employment & joint working

The survey of practices was performed in the period April to September 2015 by contacting the members of the ‘European Social Network’ (ESN), which brings together 125 public social services organizations in 34 European countries. Participating members completed a template based on seven open questions formulated in line with the nine clusters of the DMIC (see appendix I). The template also included contextual questions e.g. to describe the relevant policy, triggers for integration, and sustainability of the practice. Of the 47 templates received, 44 were sufficiently complete to be included in the study. In the remaining three templates, the answers were either incomplete or missing. An overview of the literature included and practice examples is presented in Appendix II.

The data was coded using the DMIC clusters. All data was coded using a key based on the clusters of the DMIC [9], (Appendix III). The coding was performed by SD (literature) and NZ (practices) and supervised by MM and HN. SD and NZ continuously reviewed each other’s codes. Any ambiguities and contradictions were discussed until consensus. Any discrepancies between SD and NZ were resolved by discussion with MM and HN. Finally, the results from the literature and practice reviews were compared and analysed per cluster of the DMIC by SD and NZ and reviewed by MM and HN.

## Findings

The reports from practices and articles revealed comprehensive service integration across sectors (see Table 2). The scope of the literature findings was narrow; most studies focused on health and social services integration, and there were only a few examples of integrated services involving one or more other sectors. The practice review provided a broader picture: it not only demonstrated that service integration occurs across Europe, but also that various combinations of sector integration are in place. It also demonstrated that the DMIC was applicable as a descriptive tool for service integration between the social domain and other sectors.

**Table 2:** Integration across sectors in scientific articles and practices.

Social services and		Articles N = 34	Practices N = 44

One sector	Health	21	8
	Education	–	3
	Employment	1	3
	Other	–	2
	Subtotal	22	16
Two sectors	Health & education	4	4
	Health & employment	2	–
	Health & other	2	4
	Education & other	–	2
	Subtotal	8	10
Three sectors	Health & education & employment	2	3
	Health & education & other	1	6
	Health & employment & other	–	2
	Education & employment & other	–	4
	Subtotal	3	15
Four sectors	Health & education & employment & other	1	3
	Subtotal	1	3
	Total	34	44

The literature search identified studies from a limited number of countries. The majority came from English speaking countries (see Table 3). This may be partly explained by the fact that only articles in English in peer-reviewed journals were included. The practices provided more examples of the current state of play across different European countries.

**Table 3:** Examples of service integration across sectors in included scientific articles and practices.

Country	Articles (N = 34)	Practices (N = 44)

Belgium		3
Bulgaria		1
Denmark	4	3
Finland		6
France		4
Germany		2
Greece		1
Hungary		2
Iceland		1
Italy		3
Netherlands	2	2
Poland		1
Romania		1
Slovenia		1
Spain	1	5
Sweden	5	3
United Kingdom*	9	5
-England	10	
-Northern Ireland	1	
-Scotland	3	
-Wales	2	
Total	37**	44

* 9 articles only mentioned UK, no further specification.

** Three of the articles discussed articles in multiple locations (1 × The Netherlands and Denmark, 1 × England and Scotland, 1 × England and Wales).

To determine whether integration across sectors can be analysed by the DMIC, statements relating to the nine clusters were coded in the publications included (see Table 4). The frequency with which the cluster elements appeared in the articles suggests that the DMIC describes features of service integration across sectors that are relevant to those used in practice.

**Table 4:** Reported clusters in articles.

Cluster	Present	

Inter-professional teamwork	97%	33
Delivery system	94%	32
Transparent entrepreneurship	91%	31
Person-centeredness	85%	29
Commitment	76%	26
Roles & tasks	62%	21
Performance management	56%	19
Quality care	50%	17
Result-focused learning	29%	10

Given that the practice template was based on the DMIC, each cluster was discussed in nearly all of the responses. The clusters appear to have analytical value beyond the field of health care. To investigate this finding, the clusters described below were also analysed separately for the literature review and the practices review. Since the cases reported in the literature and the practice responses were described in varying levels of detail, the analysis below presents the findings at an aggregated level and does not contain specific details of the various cases and practices.

### Person-centeredness

Literature review. Most of the examples reported in the literature aim to tailor their services to a particular target or client group. Practitioners aim to take a ‘holistic’ and ‘empowering’ approach to service delivery, meaning that the person is perceived as a partner in service delivery and is closely involved in the process [14,15]. Interestingly, this holistic approach can also include focus on a community or group of services users. Eight out of 34 services do not target a particular group, but a geographical area in order to improve the overall health of a community.

There are advantages associated with tailoring a service to an individual as well as to a community or group, as shown by user satisfaction [14] and wellbeing [16,17]. Examples of actions that are helpful in pursuing this aim include defining a clear target group [18], creating connections between different sectors [19], and between members of a community and practitioners [20,21]. However, tensions may exist between different goals of a practice, for instance, between practitioner coordination and service user involvement [22].

The literature also indicates that even when services intend to be person-centred, in reality, this may not be achieved. This may be due in part to the interests of an organization, such as set targets for volume and costs. These targets may be contrary to the interests of service users, such as practitioners working in a responsive way [23,24]. Power-related issues embedded at a societal level may also prevent implementation of a person-centred practice, for instance between policy makers and community representatives from disadvantaged areas [25].

Practice review. Most of the organizations that completed the template describe ways in which their practices aim to facilitate person-centeredness: 68% of the practices provide targeted, clear and relevant information to their service users and to families. 64% of the practices contact their users personally, by telephone or through face-to-face conversations. For instance, the Recovery practice in Denmark [P12], a project that brings together people with mental disabilities, has an emergency phone line so that service users can get in touch directly with their caregiver.

However, communication is just one aspect of person-centred service delivery and the practices describe the personalization of work or care plans as more far-reaching. In fact, 39% of the practices create personalized plans based on the unique situations of the service users and their families. In 71% of these examples, service users are involved in the design of their own plan. Service users actually co-produce the services with the professionals involved through such activities as shared decision-making about interventions. For instance, the Youth Guarantee Scheme in Pas-de-Calais in France [P15], a practice that guides vulnerable young people to personal autonomy, assists service users to set their own goals and devise their own action plan. This self-made personalised career plan forms the starting point of their training programme towards entering the labour market.

Taking a step further, some practices involve service users in the design and implementation of their integrated services. In 16% of the practices, regular input and feedback sessions are organized with service users. Meetings are held in 16% of cases, but social media may also be used (5% of total). In the Kent Integrated Care Pioneer Programme [P23] in the UK, professionals involve service users by organizing focus groups to discuss changes implemented. The professionals on the programme also communicate with users through tweet chats on Twitter, to promote learning and engagement.

Comparison. Both the literature and practice review indicate that integrated services often set out to design a person-centred practice (mentioned in 85% of the literature and 68% of the practice examples) and aim to meet certain needs of a specific target group. In addition, they customise service plans to individual requirements, and in a number of cases actively involve service users in designing and implementing service delivery at individual and group levels.

### Inter-professional teamwork & roles and tasks

Literature review. Since integrated service delivery involves collaboration between organizations from diverse sectors, inter-professional teamwork is essential, and indeed teamwork was mentioned in all of the articles. The only article that does not specifically discuss inter-professional working is by Smith et al. [18], who describe the practice from a users’ point of view. From an integration perspective, inter-professional teamwork is key, since it is not only professional and organizational boundaries that need to be crossed, but sectoral boundaries as well [25,26].

Although it is advantageous to have a diversity of actors involved in an integrated service, the different organizational and sectoral backgrounds can also make the process challenging. Being involved in a joint practice may improve relationships and communication between practitioners across sectoral boundaries, thus increasing the options available to service users [27,28]. At the same time, increasing the number of sectors involved also complicates the decision-making process and makes a service less flexible [29]. Moreover, even if collaboration is intended, this does not necessarily translate into action; the people involved may be reluctant to change their working methods [30,31,32]. When the actors do collaborate, other clashes can emerge, such as diverging interests of the practitioners involved, which may lead them to prioritize the goals of their own organization above those of the team [15,25,33]. Being involved in a collaborative practice can also lead to practitioners being excluded by their own organization, creating a perception of “us and them”, for instance, if conflicts emerge while trying to implement new methods in the home organization [17].

The literature indicates that it is essential to consider relational and communicative aspects when establishing inter-professional teamwork. Actors may be reluctant to engage in an integrated practice if they fear the erosion of their ‘own’ role [34] or if are not allowed to use their complete skills set [35]. Moreover, in addition to a lack of knowledge and understanding about each other’s role [17], practitioners may struggle to make sense of their own roles, for instance due to lack of clarity or conflicting expectations within the new environment [14,23,24].

The literature also describes measures to cope with the complexities involved in establishing inter-professional teamwork. In five articles, co-locating collaborating professionals is mentioned as helpful for inter-professional working, as it increases social interaction, relationships and trust [19]. It can also enhance opportunities for communication and information exchange [26,27,34,36]. In another example, clarification of the processes used in the practice also clarified the roles within these processes [37]. The practice may decide to appoint a ‘champion,’ who is responsible for coordinating the practice and addressing any difficulties that arise when working together [30,38]. Another approach is the creation of a virtual team designed to improve both communication and understanding about different professional roles [26]. It is also noted that organizing inter-professional teamwork and adapting to changing roles takes time [34,39].

Practice review. Professionals work together in different ways, depending on their aims and target groups. However, also some common working concepts can be seen in the practices. Firstly, in many cases professionals follow a case-management approach (48%), often supported by tools and agreed working methods. An example of this comes from Northern Ireland [P43], where a health or social care professional completes the assessment of frail older people and assigns a ‘key worker’ who functions as the single contact person for the service user. This person also co-ordinates the actions identified in the assessment. Secondly, in 32% of the cases reviewed, professionals work in multidisciplinary teams. For instance, in the Icelandic home services practice [P26], health and (public) social care professionals work together in planning home care for the elderly. Taking this a step further, in the Finnish Clubhouses model [P5] the service users themselves are part of multidisciplinary teams, where they play a role as ‘laymen’ experts. Finally, 32% of the practices studied have located all of their services in one building. The youth employment agencies in Germany [P29] are an example of this type of one-stop-shop approach. Generally, the respondents from the practices also highlighted the complexity of inter-professional working. In the templates, practitioners also described difficulties related to social and organizational factors, such as different professional cultures, unclear definitions of tasks and roles, and a lack of formal arrangements [P7, P11, P33].

Comparison. Most examples of inter-professional teamwork described in the literature and in the practices were based on collaboration between professionals from different organizational backgrounds. Organizing inter-professional teamwork is not always a question of developing top-down technocratic arrangements for structuring teams. Instead, the focus is on creating opportunities for personal interaction. For instance, the literature shows that even if collaborative working is imposed, it may not actually take place and practitioners may continue to work in their organizational or sectoral silos. In the practices review, respondents stress the complexity and pressures involved in working both at the team level and across organizational boundaries. The literature and the practices indicate that conflicts between professionals about which goals the services should prioritize often hamper integration and collaboration. Conflicting organizational and personal interests also present a challenge.

### Delivery system

Literature review. In the previous section it was stressed that communication is a vital component for successful integration across sectors. To facilitate communication, the collaborating organizations may work with shared protocols [37], joint coordinators [14], shared care plans [24], or shared assessments [26,40,41]. The increasing use of Information and Communication Technology (ICT) devices may result in new difficulties e.g. due to computer systems which may be incompatible or absent [26,34,35,38]. There are also privacy considerations to be dealt with. Even if electronic systems are compatible, privacy legislation may prohibit the sharing of personal information among different practitioners [24,29,38,42] for reasons of confidentiality. The literature also shows that procedures can easily become overly bureaucratic; this imposes a burden on the practitioners involved and can impact the quality of services [23,30,43].

Practice review. Concerning shared delivery systems, practices cite communication and ease of sharing information as important facilitators for service integration. Respondents underline the importance of ensuring that the professionals involved have quick and easy access to all the information they need. The practices recognize the growing relevance of ICT and ‘access management’ in this respect. Shared information systems may present increased opportunities for delivery of services by practices. An example is ALBORADA in Spain [P41], which has developed a tool which permits professionals from a specified region to access, exchange and share relevant information about service users’ records. This helps to ensure greater continuity of care and also means that service users no longer have to keep repeating their case histories.

Comparison. With respect to the more structural elements of integrated service delivery, the literature in this review indicates that optimal communication flow is imperative and that ICT is becoming particularly important in the provision of integrated services. Conversely, shared ICT may present challenges to the flow of information, due to incompatible computer systems or obligations to comply with privacy legislation. The practices review stresses the importance of ‘access management’ and emphasizes the importance of communication as a vital component of the delivery system.

### Performance management & Quality of care

Literature review. Performance management and quality of care parameters were not discussed in half of the literature reviewed. There are studies, however, in which the needs of the service users were measured, e.g. using standardized assessment scales [14,24,41]. The design of the service can be described as ‘theoretically informed’ [44] or ‘well-planned’ [18], without further clarification. In most of the articles, however, the design of the practice is not discussed. The challenges of performance management may be mentioned. e.g. the need to improve the quality of a practice, while simultaneously coping with budget cuts [23]. In terms of monitoring performance, the reviewed literature provides few details; practice audits [43] or evaluations [15,28,39,45,46], are usually mentioned but without providing details about how they were carried out. The use of standards and measurements may also be perceived as problematic. Articles refer to the time-consuming nature of filling out records for measurement purposes [23,42] or the difficulty in finding adequate measurement indicators to implement monitoring instruments in the first place [30,31,44]. The fact that performance management and quality of care programmes are not widely reported, does not necessarily preclude their existence. The authors may simply consider them to be irrelevant to the understanding of the case.

Practice review. In 93% of the practices, progress or performance is monitored. However, it should be noted that practices use a wide range of methods and it is not always clear exactly which parameters are being measured. For instance, some practices use multiple (validated) methods for both internal and external audits. An example of multi-method evaluation is described by the Finnish Kotitori practice [P13], a one-stop-shop for services for the elderly. This practice uses annual surveys of service user satisfaction together with feedback from individual customers to measure service users’ outcomes. Additionally, Kotitori actively involves external partners in the evaluation of the service, e.g. an audit was conducted in 2014 by KPMG which focused on the cost-effectiveness of this practice. Other practices reported that they monitor their performance through customer satisfaction surveys or in a more informal way, such as unstructured conversations with service users.

Comparison. Performance management is inferred in the literature, but there is little information about how it is carried out. Although it was not discussed in the publications reviewed in this study, practitioners who filled out the templates almost always referred to monitoring and evaluation as being common practice. Although a wide range of measurement tools are described in the practice review, it often remains unclear which parameters are actually being measured and the measurements reported are rarely based on validated instruments.

### Result-focused learning

Literature review. This cluster is mentioned least of all in the literature reviewed. Elements related to result-focused learning are discussed in only one third of the papers. Lack of training is cited in the literature as a shortcoming. As a result practitioners may feel anxious about taking on a role for which they have not been trained. [34]. Training sessions and seminars were cited as the most common learning environments used to prepare professionals for new ways of working [29,38,43,47]. Feedback sessions between collaborating actors may also be used to promote learning [46,47]. Inter-professional working can be perceived as a learning experience in itself, because it is possible to learn from each other more easily in this context, particularly if strong interpersonal relationships and networks are in place [19].

Practice review. In the practices surveyed, professional learning is frequently mentioned as a catalyst for service integration. Service integration often requires the development of new skills and training to prepare professionals for working in a new setting (e.g. working in different types of inter-professional teams). Alternatively, practices may organize team meetings and feedback sessions between professionals to share experiences and encourage learning. Going a step further, some practices mention the incorporation of learning in their work processes to establish a continuous learning and feedback cycle e.g. the French Youth Guarantee Scheme practice [P15] – a practice that provides guidance for vulnerable young people on the way to personal autonomy.

Comparison. Reflection and training or feedback activities are mentioned in the literature as ways of facilitating learning. The practices analysed describe frequent learning activities ranging from sharing experiences to training sessions for feedback and reflection. In general, learning is considered to be a contributing factor towards the development of integrated service delivery. Inter-professional working can even be regarded as a learning experience in itself.

### Commitment

Literature review. Information about the commitment of the stakeholders involved is often lacking or is only inferred from the literature reviewed. Any dissension around inter-professional teamwork and commitment becomes particularly evident when the various collaborating organizations have priorities that may clash with the goals of the service [26,32,38]. A factor which also has an impact on commitment is the potential lack of resources. For instance, competing pressures on the time available can place unreasonable demands on managing the process or participating in an integrated service [33]. Another issue is staff turn-over and the corresponding shortage of staff, discontinuity in service provision, or reliance on temporary workers who may be of limited suitability for the job required [27,34].

To ensure commitment, the reviewed literature suggests building on trust and relationships between the actors and securing agreement on clear goals at all levels of the organization [29,30,33]. In addition, strategies for increasing commitment should be tailored to a particular group in the practice, since what works at the strategic level will not necessarily apply at the tactical or operational level [33].

Practice review. Commitment is also reported as being of fundamental importance in the practices. Its importance at both the organizational and professional level is highlighted. For instance, in the Belgian Stay on Track practice [P2], i.e. a central helpdesk that provides support for young people in secondary education at risk of dropping out, all the participating organizations are committed to their shared project because they firmly believe in the partnership and its aims. The respondents mention that commitment is one of the key issues for success and sustainability of Stay on Track. Additionally, practices frequently mention that the commitment of professionals, without whom service delivery would be impossible, is at least as important as organizational commitment. It is important for professionals to have a clear understanding of the reason behind integrating services before they acknowledge that spending time on the process is a good investment.

Comparison. Service integration would not take place without the commitment of the stakeholders involved. However, collaboration often adds to the workload of professionals. It needs to be clear to the different stakeholders why it is important to spend time on collaboration and communication. The literature indicates that often the purpose of integration is not clear to those expected to implement the integrated practices. Allocating time for collaboration therefore becomes a lower priority for them. The practice review presents a similar picture; practitioners indicate that organizations only join projects when they believe that integration is better for both the service users and the organization concerned.

### Transparent entrepreneurship

Literature review. The cases examined in the literature show that managerial support as well as good leadership in the practice is essential [17,26,29,42,43,44,47]. Challenges that may arise in running a practice include time constraints (particularly when the leadership role is in addition to other responsibilities) [34,38] and potential differences in authority between the collaborating organizations [15,30,39].

Considering the finances of these cases it is clear that many practices operate in an environment where sustainable funding is limited or absent [17,31,32]. In addition, practices may need to deal with cutbacks [26,34,47]. In circumstances such as these, services may become increasingly reliant on volunteers [21]. The literature also indicates that joint commissioning may be a preferred financial arrangement within the service [30,41].

The allocation of time, and other resources to test new ways of working promotes innovation in integrated service delivery [17,47]. However, even when new or more innovative methods are discovered, it may be difficult to tailor these to existing organizations, because of the different standard operating procedures in place as well as difficulties with being consulted in the home organizations [17]. Practices can even be perceived as driving innovative behaviour through collaboration [35]; a wide range of professional skills are required in this setting.

Practice review. Leadership is frequently mentioned as a success factor for integrated service delivery. The majority of practices (75%) are led by a single public organization, usually a department in a local authority or regional government. For example, the Polish practice “Interpersonal communication in the family and at school” [P32], which organizes workshops and training courses for primary and secondary school students and their parents to support families, is fully funded by a regional governmental organization: the Lower Silesia Province. In other practices (25%), leadership is shared between different agencies. Mostly, these practices are involved in larger collaborative arrangements, like the Danish Recovery practice [P12] in which several municipalities, regional agencies and user organizations participate. In some practice examples, tasks are horizontally divided across the different stakeholders. In others, policymaking is undertaken at national level and implementation at local level.

The practices are financed using different sources and arrangements for funding. Forty-five percent are funded by joint or pooled funding of two or more organizations. In practice, many different variations in joint funding models are used. For instance, local public budgets may be combined with international foundation funds as in the case of the Curcubeu centre, Romania, [P36]. Alternatively local budgets may be combined with national budgets as in the case of Byström youth services, Finland [P3] or public budgets from different sectors, such as health, employment welfare and education may be combined (Galician network for early intervention, Spain, P35). Thirty-four per cent of practices are funded by a single agency. In most cases, this agency is a public organization or some form of inter-organizational collaboration. An example is the Danish Karriereplaner case in Horsens [P27], which is completely financed by this local authority. In 20% of practices, funding is arranged using existing resources, e.g. the health and social care access point in Bolzano, Italy [P16] where staff time is provided by the current organization and no new investment is required for this initiative.

Practices often mention that they give professionals the chance to innovate by providing time and resources for improvement and experimentation. An example of this approach is the British practice ‘Inter-professional developments between general practice and adult social work teams’ [P17] where professionals are specifically given time to explore, understand and challenge each other on their working practices. According to this practice, these activities facilitate innovation.

Comparison. Both the literature and the practice review show that organizational support and guidance are key factors in the implementation of an integrated service. In the practice review, most practices were run by a single organization. The literature emphasizes the importance of collaborative leadership or the involvement of individuals with a multi-disciplinary background. While the literature and practices highlight the important role of facilitating practitioners in pursuing integrated ways of working, the literature review indicates that insufficient support and guidance can easily impede integration. Both the literature and practice review show that it is important for professionals to have autonomy and resources to test new ways of working as well as to explore, challenge, and learn to understand each other in the workplace.

## Discussion

Service integration has become a central issue in public service delivery and professionals and service users are calling for enhanced integration of services to meet multiple and complex needs of vulnerable people. The aim of this paper was to study service integration – in different forms – between social services and health, employment and education services by analysing peer-reviewed literature and practice reviews.

The findings from the practices in particular show that service integration across sectors exists in a range of forms in a significant number of European countries. Social services collaborate with health care in particular, but often this collaboration extends to other sectors. The data from the practices showed more multi-sector integration than the literature. This finding was to be expected since the respondents to the survey were selected by design. Because of the explorative character of this study no conclusions about the distribution of cases across countries can be drawn. However, it is evident that service integration across sectors is wide-spread across Europe.

Secondly, the DMIC can be applied as a conceptual framework for service integration across sectors. This was demonstrated by the high incidence of clusters and elements that were reported in the literature (see Table 4). The clusters were referred to in the majority of the articles reviewed, with the exception of result-focused learning (29%) and quality care (50%). Similar results were found in the practice review. This implies that the DMIC can be used as a framework to analyse service integration across sectors. In addition, consistent use of this framework can improve conceptual clarity with respect to reporting studies on cross-sector integration [1].

Three major issues for further discussion were identified. These relate to person-centeredness, monitoring and evaluation, and transparent entrepreneurship. Firstly, different types of “person-centeredness” are evident in the literature. Service integration tended to focus on smaller groups of people who are coping with complex multiple problems, however, the review identified a second type of service integration that takes a holistic view and addresses the well-being of communities as a whole. Service integration in both cases involves multiple stakeholders, including service users and members of the public.

With respect to “monitoring and evaluation” the practice review shows that although this is prevalent in service delivery across sectors, it is not yet firmly established. Appropriate measures and measurement tools are not readily available. The literature review also indicates a critical stance towards measurement in general. There are differences in opinion about which measurements are appropriate and there is debate about whether the effect of a practice can be measured in isolation at all [44]. Measurement indeed becomes more challenging when collaborating across sectors, since stakeholders may have conflicting interests and the interpretation of values and outcomes may not be uniform. Practices also have different stakeholders to whom they are accountable, and often the parameters monitored are different. A range of different ICT systems, which are often incompatible, make data collection even more complex. New ways of measurement that are responsive to a diversity of stakeholders and perspectives and take the quality of the process as a standard therefore are required, rather than predefined benchmarks on outcomes and structures [48].

The third issue to be discussed addresses leadership (in DMIC terminology: transparent entrepreneurship). Given the emerging interest in autonomous teams [49,50], the emphasis on organizational support and leadership was frequently mentioned in this research. In both the literature and practice reviews, leadership is described either as an element of success or crucial to its achievement. This is in line with the umbrella review carried out by Winters et al. (2016), who describe good leadership as fundamental to integrated service delivery. However the definition of what constitutes “good” leadership is not yet clear in this context [1].

### Limitations

The literature search yielded mostly studies from the UK and publications that describe cases of integration between social and health services. These findings may be biased by one of the selection criteria, namely that articles had to be published in English. Based on the practice review, it appears that there are several examples of service integration across sectors in many more European countries and in a wider variety of forms than the scientific publications suggest. Based on this study and the methods used, no conclusion can be drawn about trends per country or about integration between specific sectors in quantitative terms. However, taking into consideration the explorative character of the study, the 34 articles and the 44 practice responses studied did provide sufficient data to highlight trends and facilitators in social service integration. This was the primary aim of the study. Also, the practice review did not find significant differences in integration between different sectors or countries, which may be due in part to the limited number of practices per ‘type’ of integration on which data was gathered and analysed.

Furthermore, a possible bias in the sampling of practices studied in the review should be noted. All practices selected are members of ESN. ESN has a large outreach in Europe, which may have increased the likelihood of responses to the template. However, using this sampling technique, non-members are not included in the practices review. This limitation does not hold for the literature search. It should also be mentioned that practitioners completed the practice templates themselves. Their perspective may have been influenced by their central role in integrated service delivery.

Another potential for bias is the inclusion of a high number of cases in which social services were collaborating with health services. Since the DMIC was developed in the health sector, this may have influenced the fit of the model to the data. Nevertheless, in the cases where the health sector was not involved or it was just one of several social service partners, the model also seemed to fit well.

### Future research

The findings of this study suggest that service integration of social services with health, education and employment services exists in many European countries. Currently research that extends beyond a single sector is limited. As it is increasingly acknowledged that human well-being and health are dependent on factors outside separate domains [4,5], service integration across sectors in the public domain deserves more scientific attention. This research indicates that the DMIC is an appropriate analytical framework in this respect, however it is recommended to use this instrument to study further examples of service integration across sectors to validate its applicability in more detail. Future research could also involve a cross-case comparative analysis of additional cases, for instance, to explore whether the DMIC is a suitable model for studying the integration between non-health sector services.

To get a better understanding of the underlying concepts, processes and mechanisms of integration across sectors, case studies employing qualitative, interpretative methods can be helpful. These studies may help elucidate how multi-level and cross-sectoral integration works, looking at the functional and normative aspects of integration [11]. Although the present paper reveals a number of generic elements of service integration across sectors, comparative studies may clarify to what extent characteristics of national and/or regional policy systems or welfare models influence service integration.

## Conclusion

This study is a preliminary exploration of the current status of service integration in Europe from a social services perspective. On the basis of current publications and practice surveys, the analysis shows that most data is available on the structure and process of developing integration rather than on outcomes. Services integration as described in this study is implemented in Europe in a variety of forms aimed at a variety of target groups and, in a number of cases, populations. In addition, this study explores potential parallels between the information reported in scientific literature on service integration and that highlighted in a survey of practices involved in service integration.

There were no substantial differences in terms of concepts, processes and mechanisms between integrated social service delivery described in the literature and in the practices surveyed. However, the practices did present a larger number of integrated services and a wider variety of arrangements in place across Europe than the literature would suggest. Future research into service integration could further explore the differences and similarities of service integration between different sectors from a social services perspective. In exploring whether integration can be understood by similar concepts, processes, and mechanisms across many levels and sectors, this analysis demonstrates that the principles of the DMIC model (which was originally aimed at modelling integration within the health sector) can also be applied to service integration across sectors. Further in-depth case studies could deepen our understanding of the complex processes involved in service integration across sectors and multiple disciplines.

## Additional Files

The additional files for this article can be found as follows:

10.5334/ijic.3107.s1 Appendix I Practice template.

10.5334/ijic.3107.s2 Appendix II Overview of included literature and practice examples.

10.5334/ijic.3107.s3 Appendix III Data structure.
