# Supplementary material for: Service Integration Across Sectors in Europe: Literature and Practice
Source: Int J Integr Care. 2018 Apr 19;18(2):6. doi: 10.5334/ijic.3107 (PMC6095054; doi:10.5334/ijic.3107)
Supplement: Appendix I — Practice template. [file ijic-18-2-3107-s1.pdf]

Appendix I. Practice template

Practice template: Integrated Services

1. DETAILS CONTACT PERSON	
Name	
E mail & phone number	
Organisation	
Country, City	

2. COUNTRY DESCRIPTION	
Which policies and/or legal frameworks affect the integration or cooperation of services? If so, in what way: stimulating or limiting?<br><br>*Elaborate on relevant legal framework and policymaking regarding the following levels:*<br>*(Maximum 250 words per level)*	
What is helpful or is a barrier regarding the national system?<br>*Elaborate on elements of the national system that are relevant to this practice.*	

3. DETAILS PRACTICE		
What is the name of the practice?<br>*Provide the name both in your own language and a translation into English*		
How would you summarise the practice?<br>*Give a short description of the integrated practices example (Maximum 250 words)*		
Which phase-description suits the current status of the practice?<br>*For a detailed description, see the background paper. Tick the box that applies to your practice, and if applicable, indicate any differences between the description and the actual practice execution.*	Initiative and design phase	
	Experimental and execution phase	
	Expansion and monitoring phase	
	Consolidation and transformation phase	

4. PRACTICE START-UP	
Why was it necessary to develop this practice?<br>*Elaborate on what triggered the start of the practice, and also elaborate on why an integrated services' approach is chosen.*	
What is the main aim of the practice?<br>*Elaborate on the expected output in the new situation compared to the old situation.*	
Who is the target group for the practice?<br>*Please specify the age group and socio-demographic characteristics.*	
What problems does the target group face?	
How does the practice fulfil the target group's needs?<br>*Elaborate on how the stakeholders deliver integral support*	

(e.g. the actions that are carried out).	

5. COLLABORATION STRUCTURE	
On which level does the integration of public services take place: country/county/region/municipality?<br>*Elaborate if possible.*	
Which sectors (social services, health, education, employment /organisations/ professionals) are cooperating with each other?	
Which organisations are collaborating with each other?<br>*Name all the stakeholders involved.*	
Who is leading the initiative and why?<br>*Specify the person, function, and organization.*	
How is the practice financed?<br>*Elaborate on the financial structure (budget) and indicate whether or not funding is used.*	
How is information regarding service users exchanged between the collaborating service providers?<br>*Elaborate on aspects like care plans, decision-making, and record keeping.*	
How do service providers communicate with service users?<br>*Elaborate on aspects such as access to care and information via for instance contact persons.*	

6. PRACTICE EVALUATIONS	
Was the practice itself based on an already existing example?<br>*Please elaborate on the origin of the practice/idea and explain to what degree, if any, the practice uses evidence-based guidelines and standards.*	
How are the effects/ results of the practice measured?<br>*Which instruments, infrastructure, and indicators are used for measurement?*	
What were the main effects of the practice?<br>*Elaborate on the impact that the practice has/had on the following groups.*<br>*Please indicate whether you perceive the effect as positive or negative.*	Service users:<br>Their families:<br>Professionals:<br>Organisations:
What were the unexpected effects of the practice?<br>*While answering, indicate whether you perceive the effect as positive or negative.*	
Do you see potential to expand the practice?<br>*Elaborate about the ability of the practice to be scaled up, implemented in different places/settings (e.g. other sectors, cities, countries) and explain why you think so.*	
What do you perceive as the strengths of the practice?<br>*Elaborate on **why** you regard these to be strengths.*	
What do you perceive as the weaknesses of the practice?<br>Elaborate on why you consider these to be weaknesses.<br><br>Which lessons can be learned from this practice according	

to you?	

REMARKS
Do you have any remaining remarks, questions, suggestions that are not included in the questionnaire?

ATTACHMENTS
Do you have any relevant documents that can be used by ESN (and Vilans) to analyse the practice further?
Links:<br><br>Attachment:
