# Supplementary material for: Service Integration Across Sectors in Europe: Literature and Practice
Source: Int J Integr Care. 2018 Apr 19;18(2):6. doi: 10.5334/ijic.3107 (PMC6095054; doi:10.5334/ijic.3107)
Supplement: Appendix II — Overview of included literature and practice examples. [file ijic-18-2-3107-s2.pdf]

Appendix II. Overview of included literature and practice examples

Table I. Overview results literature review

Article	Method	Country	Domains	Program name	Target group
Belling et al. (2011)	Mixed methods	England	Social, health	Community mental health teams (CMHTs)	Adults
Carlisle (2010)	Qualitative	Scotland	Social, health	Social Inclusion Partnership (SIP)	Community
Collins & McCray (2012)	Qualitative	United Kingdom	Social, health, education	Common Assessment Framework (CAF)	Children
Davidson et al. (2012)	Mixed methods	Northern Ireland	Social, health	Champions Initiative	Children, adults
Devanney & Wistow (2013)	Quantitative	England	Social, health, education, police	Children's Trusts	Children
Durie & Wyatt (2012)	Qualitative	England, Scotland	Social, health	Connecting Communities (C2)	Community
Edvardsson et al. (2011)	Qualitative	Sweden	Social, health, education	Salut Programme	Children
Germundsson & Danermark (2012)	Qualitative	Sweden	Social, health, employment	New ways to the labour market and quality assurance of cooperation	Adults
Golding (2010)	Qualitative	England	Social, health, education	Integrated Service for Looked After and Adopted Children (ISL)	Children
Goodman et al. (2011)	Quantitative	England	Social, health	Interprofessional Working (IPW)	Elderly
Green & Dicks (2012)	Qualitative	United Kingdom	Social, health	Private case management	Adults
Hall & McGarroll (2013)	Qualitative	United Kingdom	Social, health, education, employment, housing	Local Area Coordinators (LAC)	Adults
Hansson et al. (2010)	Qualitative	Sweden	Social, health	Mental health & Social care consortium	Adults
Hansson et al. (2012)	Qualitative	Sweden	Social, health	Mental health & Social care consortium	Adults
Hendriks et al. (2012)	Qualitative	The Netherlands	Social, health	Integrated Enabling Policies	Children
Hunter & Perkins (2012)	Qualitative	England	Social, health	Public Health Partnerships	Community
Kellehear (2013)	Quantitative	England	Social, health, education,	Compassionate Communities	Community

			employment		
King et al. (2012)	Qualitative	Scotland	Social, health	Electronic Single Shared Assessment	Elderly
Mahmud et al. (2010)	Quantitative	Sweden	Social, health	Health Square (HS)	Community
Manthorpe & Martineau (2012)	Qualitative	England, Wales	Social, health	Serious Case Review	Adults
Molina et al. (2013)	Mixed methods	Spain	Social, health	SAIATU program	Adults, elderly
Petch et al. (2013)	Qualitative	United Kingdom	Social, health	Health and Social Care Partnerships	Adults, elderly
Pittam et al. (2010)	Qualitative	United Kingdom	Social, health, employment	Condition Management Programmes (CMPs)	Adults
Richardson et al. (2013)	Qualitative	United Kingdom	Social, health	Stepping Stones to Nature (SS2N)	Community
Rudkjobing et al. (2014)	Quantitative	Denmark	Social, health	Health Care Agreements	All
Sestoft et al. (2014)	Qualitative	Denmark	Social, health, justice	PSP model	Adults
Smith & Barnes (2013)	Qualitative	United Kingdom	Social, health, third sector	Partnerships for Older People Projects (POPP)	Elderly
Smith et al. (2013)	Mixed methods	Denmark	Social, employment	Make a Difference	Adults
Svendsen (2010)	Qualitative	The Netherlands, Denmark	Social, health, education, employment	Multifunctional Centers	Community
Taylor-Robinson et al. (2012)	Qualitative	England	Social, health	Local Strategic Partnerships (LSPs)	Diverse examples
Watson et al. (2014)	Quantitative	United Kingdom	Social, health, education	Sure Start Children's Centres	Children
Webber et al. (2013)	Quantitative	United Kingdom	Social, health	Multi-agency joint protocols	Children
Wilberforce et al. (2011)	Quantitative	England	Social, health	Community mental health teams for older people (CMHTsOP)	Elderly
Williams (2012)	Qualitative	Wales	Social, health	Community Integrated Intermediate Care Service (CIIS)	Community

Table. Overview of practices

Ref.	Country	Name of Practice	Target Group	Domains
1	Belgium	*Children First: Local Consultation Platforms for Prevention and Identification of Child Poverty I*	Children and families	social services, education, other
2	Belgium	*Stay On Track / Central Helpdesk*<br>*Dropout prevention network with central helpdesk Youth at risk.*	Young people	social services, education, employment, health, other
3	Finland	*Byström youth services*	Young people	social services, education, employment, health, other
4	Finland	*Hietaniemenkatu service center*	Homeless	social services, health, other
5	Finland	*Klubitalomenetelmä / Clubhouse model*	Mental Health	social services, education, employment, other
6	Finland	*Lafos The Labour Force Service Centre*	Unemployed	social services, health, employment, other
7	Germany	*Basic assistance for job seekers*	Unemployed	social services, education, employment, other
8	Spain	*Network of social support programmes FAISEM*	Mental Health	social services, education, employment, health, other
9	Spain	*Espai Cabestany*	Young people	social services, education, employment, health, other
10	Netherlands, The	*'Medical Advice for Sick-reported Students', abbreviated as MASS.*	Young people	social services, health, education
11	Bulgaria	*Community Mental health*	Mental Health	social services, health, other
12	Denmark	*Recovery approach*	Mental Health	social services, health, employment, other
13	Finland	*Kotitori - Health and social care Integrator*	Older people	social services, health, other

14	France	*Houses for personal autonomy*	Older people	social services, health
15	France	*Youth Guarantee Scheme*	Young people	social services, employment
16	Italy	*Older people access to care*	Older people	social services, health
17	UK	*Interprofessional sessions*	All target groups	social services, health
18	Sweden	*Nyborg/SKIFO*	Children and young people	social services, education, other
19	Sweden	*SIMBA*	All target groups	social services, health, education
20	Sweden	*Social Planning Group*	All target groups	social services, other
21	Netherlands, The	*Employment project for (young) people with disabilities*	Disability	social services, education, employment, health, other
22	UK	*"Boiler on prescription" pilot*	Older people	social services, health, housing
23	UK	*Kent Integrated Care Pioneer*	Older people	social services, health, other
24	Spain	*Health and social*	Older people	social services, health
25	Belgium	*Case-management*	Unemployed	social services, employment
26	Iceland	*Home services*	Older people	social services, health
27	Denmark	*Career plan*	Disability	social services, employment
28	Denmark	*Open dialogue*	Mental Health	social services, health, education, employment
29	Germany	*Youth employment Agencies*	Young people	social services, health, education, employment

30	France	*Integration Policy*	Older people	social services, health
31	Slovenia	*Helping families at home*	All target groups	social services, health
32	Poland	*Workshops at schools*	Children	social services, education
33	UK	*Working collaboratively to provide a wide range of day opportunities to adults with learning disabilities*	Disability	social services, health, education, other
34	Finland	*Family Net - The NGOs' Family Centre and Welfare Network –Model*	Children and Families	social services, health, education, other
35	Spain	*Early Childhood Intervention*	Children	social services, health, education, other
36	Romania	*Day Care Centra for Children*	Children	social services, health, education
37	Hungary	*Basic Access to social services*	Older people	social services, health, education
38	Hungary	*The signaling system*	Children	social services, education
39	Italy	*Casa Ella Project*	Children with disabilities	social services, health, education, employment
40	Greece	*FrontiZo Care*	Children	social services, education
41	Spain	*ALBORADA*	Children	social services, health, education, other
42	France	*LAEP*	Children and Families	social services, health, education, other
43	UK	*Single Assessment Tool*	Older People	social services, health
44	Italy	*Regional Integrated Program of Services for people with ASD (autism)*	Mental Health	social services, health, education, other
