# Supplementary material for: Service Integration Across Sectors in Europe: Literature and Practice
Source: Int J Integr Care. 2018 Apr 19;18(2):6. doi: 10.5334/ijic.3107 (PMC6095054; doi:10.5334/ijic.3107)
Supplement: Appendix III — Data structure. [file ijic-18-2-3107-s3.pdf]

Appendix III. Data structure

Table 3. Data structure		
Cluster	Coded segments in literature	Coded segments in practice
Person-centeredness	“Interviewees recognized the child in need as a common focus of activity; however, service –specific objectives were believed to detract from a holistic approach and competing priorities generated tensions in practice. [23, p. 136]<br><br>“other professional groups sometimes let their organization’s perspective prevail over the needs of the client.” [17, p. 513]<br><br>“a person-centred plan was used with Jake to continually identify his “felt need”. At all times Jake’s options were clearly outlined to him, reinforcing his role as a collaborative partner.” [22, p. 9]	“The service center is a small community and the clients are treated with dignity. The staff knows the clients. This helps to offer individual services. The services in one place enable a comprehensive approach to the situation of a client.” [P4]<br><br>“The Houses for personal autonomy especially through the one-stop-shop service approach aim to:<br>• Simplify the users’ path;<br>• Allow people to stay in their place as long as possible;<br>• Tailored-made, person-centred and integrated approach especially between the service providers;<br>• Enhance support for carers through the development of platforms for assistance.” [P14]<br><br>“Help the young people to acquire the necessary skills and attitudes to enter into the labour market thought training programme, advice and counselling, etc. + to develop a personalised career plan + to become self-independent.” [P15]
Interprofessional teamwork	“the majority of professionals in both organisations had positive experiences of working co-located, integrated, multidisciplinary teams and these facilitated continuity.” [34, p. 5]<br><br>“Although SIP leaders and community representatives shared an understanding that people living in disadvantaged areas needed more services and facilities than better-off areas, this failed to develop into positive collaboration because of their	“We have 4 integrated care centres in Kent, providing long and short term (intermediate care)beds. Services are delivered within these buildings by integrated health and social care staff teams.” [P23]<br><br>“Information regarding service users are exchanged in multidisciplinary teams, by phone and email.”[P31]<br><br>“Multidisciplinary teams work in the framework of an agreed convention.” [P42]

	intrinsic conflict of interest." [25, p. 123]<br><br>"The collaboration process was described in positive terms, even though rigid rules and lack of management and resources were seen to cause difficulties, especially in the relation between the individual projects and the home organisations." [16, p. 514]	
Roles & tasks	"having an identified Champion increases the risk that other members of the team may think that the Champion carries responsibility for interface issues rather than the whole team." [38, p. 168]<br><br>"a common denominator related to unclear roles and routines became dear. The ambiguity was described both in relation to internal contacts with colleagues and also associated to external contact with authorities." [14, p. 6]<br><br>"There were also issues of jurisdictional legitimacy, such as whether the completion of the electronic SSA fits with the nurses' perception of their role." [26, p. 9]	"Regrettably, very often the roles and responsibilities of the different stakeholders, related to a particular case, are not clearly defined and communicated to them, so that the client could be placed in the centre of a comprehensive support network." [P11]<br><br>"to ensure that all partners are clear as to actions, responsibilities and outcomes sound communication is essential. Styles and approaches to communication needed to be agreed by all." [P33]
Delivery system	"Practitioners also reported that the protocols had clarified the referral processes between agencies" [37, p. 153]<br><br>"inadequate provision of IT equipment was a barrier for information, flexible and long-term continuity, due to incompatibility of software systems, use of outdated computers hardware, which in some cases was shared with other professionals, and lack of finance to update provision." [34, p. 5]<br><br>"some factors have been a hindrance to the development of the cooperation, including the unclear legal framework for	"It is about providing integrated home services to those in need of assistance so they can be able to live in their home as long as they choose. Providing "one stop shop", more simple application process. People apply for services in one place and are to receive more integral services than before from nurses and home work service people. There is a cooperation and coordination between the nurses and home work people in every case, f.ex. by organising visits throughout the day in one home if necessary instead of before maybe both workers (nurse and social home services) came at the same time."[P26]<br><br>"The project created various products (e. g. cooperation agreements,

	exchange of information within the cooperation." [29, p. 373]	documents on points of intersection, labour market programmes across legal spheres) and established coordinated processes.The following approach is applied at the "One Stop Shop", which means all services are provided within one organization." [P29]<br><br>"The electronic enabler for the Northern Ireland Single Assessment Tool has been implemented Regionally across multi-disciplinary teams (Nurses, Social workers, Allied Health Professionals) in all 5 Health and Social Care Trusts in in older people's services." [P43]
Performance management	"A range of research and audit-based evaluations has confirmed that ISL provides an effective additional level of support for the children, carers and professional network." [42, p. 581]<br><br>"A weakness that could make it difficult to plan for further development of existing HSs and implementation of new ones nationwide, is the lack of standards for assessment of HS activities." [15, p. 6]<br><br>"Due to the complexity and ambitious expectations, the model ran into some significant implementation delays and by the end of the pilot, some areas had not been fully operational for long enough to assess impact." [39, p. 83]	"Evaluation done by the Ministry of Employment and the Economy (2008), Arnkil & Spangar (2008)" [P6]<br><br>"evaluation survey conducted among the participants of workshops and trainings" [P32]
Quality care	"The project team found that collating evidence for the HIA was a useful process to go through in terms of recognizing the breadth of health and well-being that the project could offer." [20, p. 509]<br><br>"Contributing to the language barrier was the difficulty of reconciling what is good evidence for an inter-sectoral intervention, because different sectors value evidence in different ways." [31, p. 4]	"Annual customer satisfaction survey, individual customer feedback and reclamations. Also monthly follow-up of the number of given guidance (phone calls, answered calls, guidance given at the drop-in centre). Also indicators for guidance given by principal "answer directly" and evaluation of service needs made in 5 days (%)." [P13]<br><br>"A specific check-list regarding abilities and autonomies has been set up in order to regularly test the level of acquired skills and abilities.

	"Although awareness of a particular problem might seem the most logical step to recognizing it, actors at the strategic level often do not base their problem definition on objective analysis, but rather use it as a strategic activity to gain support for their point of view" [33, p. 5]	Vineland scale has been used since 2014 to evaluate new entries. It allows to measure over time both acquired skills and existing difficulties." [P39]
Result-focused learning	"Where inter-personal relationships and networks are strong and stocks of social capital high, learning with, and from each other, is more likely to occur." [19, p. 555]<br><br>"Continuous learning was facilitated by sharing knowledge, advice, and ideas, and by giving and getting feedback." [47, p. 7]	"International training and capacity-building for members, staff and administrators of CHs" [P5]<br><br>"Specific education material and sessions for the local pilots, and for case managers (university degree for case managers created)" [P30]
Commitment	"The importance of goodwill was especially evident at frontline level. Allied to this feature wat the existence of 'local champions' who played a crucial role in partnerships. Their particular contribution lay in their commitment and enthusiasm which could, in turn, encourage others to commit to partnership working." [30, p. 8]<br><br>"Shared goals and shared commitment to CMP were identified by some participants as having helped to overcome initial difficulties." [35, p. 703]<br><br>"the general practitioners were more familiar with the healthcare agreements than the previous health plans and reported that they have a more significant influence on their work. Still, less than half of them experience that the health care agreements influence their work 'to some' or 'to a high degree'." [46, p. 224]	"Each partner organisation 'voluntarily' joined the network because all of them believe the client and the organisations (service providers) can benefit."[P2]<br><br>"Community that not only economically supports the project, but it also raises awareness and creates a sense of belonging."[P39]
Transparent entrepreneurship	"Many statements suggested that commitment and support from management was required if collaboration were to be successful and effective over time" [17, p. 511]	"In the beginning the initiative was funded by the Finnish Funding Agency for Technology and Innovation (Tekes).<br>Nowadays the City of Tampere covers costs related to the integrator

	“The community house was opened in March 12, 2006. Since then, half of the budget has been financed by the municipality.” [21, p. 61]	services (needs assessment and advice & service planning) provided by the Kotitori case managers. The service users pay for personal and household services apart from services funded by the municipality under specific criteria.” [P13]
	“Lack of capacity in terms of staff (34%) and lack of funding (29%) were the two most frequently mentioned barriers.” [32, p. 44]	“a common strategic vision signed up to by all parties (the national collaborative), formal partnership arrangements within each local area (health and well-being boards), sharing of budgets across health and social care (better care fund and personal health and social care budgets), national support for innovative practice (integrated care pioneers), care co-ordination for those with most complex needs (named accountable GP for people over 75) and the potential for new organisational forms (multi-specialty community providers).” [P45]
		“Pestalozzi Children`s Foundation in Switzerland was the principal funder and Arad municipality from the local budget” [P36]
